# A rapid screening model for early predicting novel coronavirus pneumonia in Zhejiang Province of China: a multicenter study

**DOI:** 10.1038/s41598-021-83054-x

**Published:** 2021-02-16

**Authors:** Yi-Ning Dai, Wei Zheng, Qing-Qing Wu, Tian-Chen Hui, Nan-Nan Sun, Guo-Bo Chen, Yong-Xi Tong, Su-Xia Bao, Wen-Hao Wu, Yi-Cheng Huang, Qiao-Qiao Yin, Li-Juan Wu, Li-Xia Yu, Ji-Chan Shi, Nian Fang, Yue-Fei Shen, Xin-Sheng Xie, Chun-Lian Ma, Wan-Jun Yu, Wen-Hui Tu, Rong Yan, Ming-Shan Wang, Mei-Juan Chen, Jia-Jie Zhang, Bin Ju, Hai-Nv Gao, Hai-Jun Huang, Lan-Juan Li, Hong-Ying Pan

**Affiliations:** 1 Department of Infectious Diseases, Zhejiang Provincial People’s Hospital, People’s Hospital of Hangzhou Medical College, No. 158 Shangtang Road, Hangzhou, 310014 Zhejiang China; 2 The Second Clinical Medical College, Zhejiang Chinese Medical University, Hangzhou, China; 3 Bengbu Medical College, Bengbu, China; 4 Hangzhou Wowjoy Information Technology Co., Ltd, Hangzhou, China; 5 Clinical Research Institute, Zhejiang Provincial People’s Hospital, People’s Hospital of Hangzhou Medical College, Hangzhou, China; 6 Key Laboratory of Endocrine Gland Diseases of Zhejiang Province, Hangzhou, China; 7 Medical College of Qingdao University, Qingdao, China; 8 Department of Infectious Diseases, First People’s Hospital of Tongxiang, Jiaxing, China; 9 Department of Infectious Diseases, People’s Hospital of Shaoxing, Shaoxing, China; 10 Department of Infectious Diseases, Wenzhou Central Hospital, Wenzhou, China; 11 Department of Infectious Diseases, First Hospital of Taizhou, Taizhou, China; 12 Department of Infectious Diseases, First People’s Hospital of Xiaoshan, Hangzhou, China; 13 Department of Infectious Diseases, First Hospital of Jiaxing, Jiaxing, China; 14 Department of Infectious Diseases, First People’s Hospital of Wenling, Taizhou, China; 15 Department of Respiratory and Critical Care Medicine, Yinzhou People’s Hospital, Affiliated Yinzhou Hospital, College of Medicine, Ningbo University, Ningbo, China; 16 Department of Infectious Diseases, Taizhou Municipal Hospital, Taizhou, China; 17 Department of Infectious Diseases, ShuLan (Hangzhou) Hospital Affiliated to Zhejiang Shuren University Shulan International Medical College, Hangzhou, China; 18 State Key Laboratory for Diagnosis and Treatment of Infectious Diseases, National Clinical Research Centre for Infectious Diseases, Collaborative Innovation Centre for Diagnosis and Treatment of Infectious Diseases, the First Affiliated Hospital, College of Medicine, Zhejiang University, Hangzhou, 310003 Zhejiang Province China

**Keywords:** Infectious diseases, Infectious-disease diagnostics

## Abstract

Novel coronavirus pneumonia (NCP) has been widely spread in China and several other countries. Early finding of this pneumonia from huge numbers of suspects gives clinicians a big challenge. The aim of the study was to develop a rapid screening model for early predicting NCP in a Zhejiang population, as well as its utility in other areas. A total of 880 participants who were initially suspected of NCP from January 17 to February 19 were included. Potential predictors were selected via stepwise logistic regression analysis. The model was established based on epidemiological features, clinical manifestations, white blood cell count, and pulmonary imaging changes, with the area under receiver operating characteristic (AUROC) curve of 0.920. At a cut-off value of 1.0, the model could determine NCP with a sensitivity of 85% and a specificity of 82.3%. We further developed a simplified model by combining the geographical regions and rounding the coefficients, with the AUROC of 0.909, as well as a model without epidemiological factors with the AUROC of 0.859. The study demonstrated that the screening model was a helpful and cost-effective tool for early predicting NCP and had great clinical significance given the high activity of NCP.

## Introduction

Since December 2019, novel coronavirus pneumonia (NCP) emerged in Wuhan, Hubei, which was well known as the largest transportation hub in China. The pathogen has been proved to be a novel betacoronavirus that is currently named 2019 novel coronavirus (2019-nCoV)^1^. The disease has swept across China rapidly through human-to-human transmission^2–4^. Since February 27, 2020, more than 78,000 people were confirmed to be infected and more than 2700 were died in China^5^.

As the number of patients soaring, scholars have summarized the clinical characteristics of NCP^6–8^. Symptoms at onset of disease included fever, cough, headache, vomiting, diarrhea and so on. Normal or decreased leukocyte count was common. Radiologic abnormalities like ground-glass opacity and patchy shadowing on chest X-ray or computed tomography (CT) were marked characteristics. Acute respiratory distress syndrome, arrhythmia and shock could also occur in severe cases. Until now, to detect 2019-nCoV by the accurate real-time reverse transcription polymerase chain amplification (RT-PCR) assessment has been regarded as the golden diagnostic standard^9^.

Nevertheless, false negative results in initial RT-PCR examination existed in a number of cases^10^. Besides, the time-consuming process, short supply of kits, and difficulty in qualified sampling prevented us from early-stage diagnosis and treatment, as well as prompt isolation of patients. Therefore, it necessitates establishment of a rapid diagnostic model to screen high-risk patients with 2019-nCoV infection.

In this study, we aimed to develop a novel screening scale to determine highly suspected subjects based on epidemiological data, clinical manifestations, laboratory and radiological examinations. Given the evolution of the pandemic, we combined the epidemic regions into one parameter, and even dropped the epidemiological factors from the model, to establish another two simplified, but still effective models. It is the first study for screening and predicting NCP in Zhejiang Province, China, and can be popularized nationwide and even worldwide.

## Results

### Clinical characteristics of the study participants

Of the 880 subjects enrolled in the study, 21 subjects were excluded due to missing data, and 859 participants were eligible for evaluation. Of them, 339 were diagnosed as NCP with the positive detection of 2019-nCoV by real-time RT-PCR, while the other 520 participants were ruled out with at least two times negative results by RT-PCR. The 21 excluded cases refused chest X-ray or CT because of pregnancy or preparing for pregnancy. Fortunately, their RT-PCR tests all showed negative results.

The characteristics of participants were exhibited in Table 1. Among these 339 NCP sufferers, 188 (55.46%) were male, and the mean age was 46.88 ± 14.65 years. The age of NCP sufferers was significantly larger than those without NCP (*P* < 0.001). 33.63% of the confirmed patients had a history of travel or residence in Wuhan within 14 days, and 27.43% had contacted patients with fever or respiratory symptoms from Wuhan within 14 days. 35.10% of the confirmed cases were related to cluster outbreaks in families or places of work.

**Table 1:** Clinical characteristics of the study participants.

	Patients with NCP (n = 339)	Patients without NCP (n = 520)	*P* value
Age (years)	46.88 ± 14.65	37.70 ± 18.03	< 0.001
Sex (male)	188 (55.46%)	263 (50.58%)	0.163
**Coexisting diseases**	101 (29.79%)	66 (12.69%)	< 0.001
Hypertension	67 (19.76%)	40 (7.69%)	< 0.001
Diabetes mellitus	21 (6.19%)	12 (2.31%)	0.006
Cancer	4 (1.18%)	4 (0.77%)	0.719
COPD	3 (0.88%)	9 (1.73%)	0.382
Hepatitis B infection	8 (2.36%)	4 (0.77%)	0.072
Others	6^a^ (1.77%)	11^b^ (2.12%)	0.806
**Travel or residence history within 14 days**			
Wuhan	114 (33.63%)	68 (13.08%)	< 0.001
Neighboring areas of Wuhan in Hubei Province	9 (2.65%)	67 (12.88%)	< 0.001
Other areas with persistent local transmission, or community with definite cases	149 (43.95%)	278 (53.46%)	0.007
**Contacting patients with fever or respiratory symptoms within 14 days who had a travel or residence history in the following areas**			
Wuhan	93 (27.43%)	64 (12.31%)	< 0.001
Neighboring areas of Wuhan in Hubei Province	8 (2.36%)	24 (4.62%)	0.097
Other areas with persistent local transmission, or community with definite cases	88 (25.96%)	81 (15.58%)	< 0.001
Relationship with a cluster outbreak	119 (35.10%)	14 (2.69%)	< 0.001
Exposure to wildlife	1 (0.29%)	1 (0.19%)	1.000
Contact with patients of influenza A	3 (0.88%)	15 (2.88%)	0.052
Contact with patients of influenza B	5 (1.47%)	15 (2.88%)	0.248
Fever^c^	162 (47.79%)	247 (47.50%)	1.000
Body temperature	37.50 ± 0.82	37.49 ± 0.83	0.895
< 37.5 °C	177 (52.21%)	273 (52.50%)	0.768
37.5–38.5 °C	109 (32.15%)	173 (33.27%)	
≥ 38.5 °C	53 (15.63%)	74 (14.23%)	
Dry cough	149 (43.95%)	194 (37.31%)	0.055
Sputum	118 (34.81%)	123 (23.65%)	< 0.001
Fatigue	90 (26.55%)	57 (10.96%)	< 0.001
Dyspnea	33 (9.73%)	11 (2.12%)	< 0.001
Conjunctival congestion	2 (0.59%)	4 (0.77%)	1.000
Nasal congestion	12 (3.54%)	44 (8.46%)	0.004
Diarrhea or bellyache	32 (9.44%)	19 (3.65%)	0.001
Dizziness or headache	28 (8.26%)	46 (8.85%)	0.805
Nausea or vomiting	12 (3.54%)	7 (1.35%)	0.054
Sore throat	19 (5.60%)	58 (11.15%)	0.005
Muscle soreness	17 (5.01%)	3 (0.58%)	< 0.001
White blood cell count (× 10^9^)	5.40 ± 2.56	7.36 ± 3.06	< 0.001
Normal or decreased white blood cells^d^	318 (93.81%)	428 (82.31%)	< 0.001
Lymphocyte count (× 10^9^)	1.22 ± 0.85	1.71 ± 0.86	< 0.001
Decreased lymphocytes^e^	184 (54.28%)	134 (25.77%)	< 0.001
Neutrophil cell count (× 10^9^)	3.93 ± 4.40	5.05 ± 3.34	< 0.001
Normal or decreased neutrophil cells^f^	302 (89.09%)	400 (76.92%)	0.531
**C-reactive protein level (mg/L)**	21.24 ± 27.06	18.88 ± 35.61	0.271
≤ 10 mg/L	159 (46.90%)	327 (62.88%)	0.001
10–50 mg/L	141 (41.59%)	140 (26.92%)	
> 50 mg/L	39 (11.50%)	53 (10.19%)	
**Chest X-ray or CT**			
Normal	16 (4.72%)	230 (44.23%)	< 0.001
Unilateral local patchy shadowing	93 (27.43%)	130 (25.00%)	
Bilateral multiple ground glass opacity	118 (34.81%)	97 (18.65%)	
Bilateral diffuse ground glass shadowing with pulmonary consolidation	109 (32.15%)	30 (5.77%)	
Other imaging alterations such as pulmonary nodule or pleural effusion	3 (0.88%)	33 (6.35%)	

*NCP* novel coronavirus pneumonia, *COPD* chronic obstructive pulmonary disease, *CT* computed tomography.

^a^One valvular heart disease, one atrial fibrillation, one HIV infection, two cases of ankylosing spondylitis, and one anxiety disorder.

^b^Three cases of chronic nephritis, two cases of cerebral infarction, one depression, one schizophrenia, one rheumatoid arthritis, one gout, one hypothyroidism, and one trauma.

^c^Fever is defined as body temperature > 37.5 °C.

^d^Normal range of white blood cell count: 3.50–9.50 × 109.

^e^Normal range of lymphocytes: 1.10–3.20 × 109.

^f^Normal range of neutrophil cells: 1.80–6.30 × 109.

The common symptoms of NCP included fever (47.79%), dry cough (43.95%), sputum (34.81%), fatigue (26.55%) and dyspnea (9.73%). But fever was not a specific symptom because it was also commonly seen in non-NCP individuals (47.50%, *P* = 1.000). Normal or decreased WBC count happened in 93.81% of NCP patients, and decreased lymphocyte count was seen in 54.28% of patients. The mean WBC count in NCP group was 5.40 ± 2.56 (× 10^9^/L), significantly lower than those without NCP (7.36 ± 3.06, × 10^9^/L, *P* < 0.001). Meanwhile, the lymphocyte count was 1.22 ± 0.85 (× 10^9^/L), significantly lower than those without NCP (1.71 ± 0.86, × 10^9^/L, *P* < 0.001). No significant difference was found between the two groups in terms of C-reactive protein level (CRP). Most NCP patients (94.39%) had pulmonary radiologic changes like unilateral or bilateral patchy shadowing, ground-glass opacity or pulmonary consolidation on X-ray or CT.

### Predictors associated with NCP

We performed both univariate and multivariate logistic regression analyses to assess predictors of NCP (Table 2). In the univariate analysis, age, co-existing diseases, travel or residence history within 14 days in Wuhan, neighboring areas of Wuhan in Hubei Province, and other areas with persistent local transmission, or community with definite cases, contacting patients with fever or respiratory symptoms within 14 days from Wuhan, neighboring areas of Wuhan in Hubei Province, and other areas with persistent local transmission or community with definite cases, relationship with a cluster outbreak, presence of sputum, fatigue, dyspnea, diarrhea or bellyache, muscle soreness, absence of nasal congestion or sore throat, decreased WBC count, lymphocyte count, and neutrophil cell count, and imaging changes in chest X-ray or CT were observed to be associated with higher odds of NCP.

**Table 2:** Predictors associated with NCP.

Variables	Univariate	Multivariate			
	*P* value	β ± SE	Wald	OR (95% CI)	*P* value
Age	**< 0.001**	0.003 ± 0.008	0.124	1.003 (0.988–1.018)	0.725
Gender	0.162				
Coexisting diseases	**< 0.001**	0.502 ± 0.297	2.853	1.652 (0.923–2.956)	0.091
**Travel or residence history within 14 days**					
Wuhan	**< 0.001**	**2.133 ± 0.356**	**35.976**	**8.440 (4.204–16.944)**	**< 0.001**
Neighboring areas of Wuhan in Hubei Province	**< 0.001**	− 0.552 ± 0.551	1.003	0.576 (0.196–1.696)	0.317
Other areas with persistent local transmission, or community with definite cases	**0.007**	0.493 ± 0.307	2.575	1.637 (0.897–2.987)	0.109
**Contacting patients with fever or respiratory symptoms within 14 days who had a travel or residence history in the following areas**					
Wuhan	**< 0.001**	**1.088 ± 0.306**	**12.663**	**2.967 (1.630–5.402)**	**< 0.001**
Neighboring areas of Wuhan in Hubei Province	0.084				
Other areas with persistent local transmission, or community with definite cases	**< 0.001**	**1.421 ± 0.292**	**23.602**	**4.139 (2.334–7.342)**	**< 0.001**
Relationship with a cluster outbreak	**< 0.001**	**3.225 ± 0.385**	**70.194**	**25.164 (11.833–53.516)**	**< 0.001**
Exposure to wildlife	0.762				
Contact with patients of influenza A	0.059				
Contact with patients of influenza B	0.189				
Body temperature	0.895				
Dry cough	0.052				
Sputum	**< 0.001**	0.203 ± 0.243	0.695	1.225 (0.760–1.973)	0.404
Fatigue	**< 0.001**	**0.997 ± 0.305**	**10.664**	**2.710 (1.490–4.930)**	**0.001**
Dyspnea	**< 0.001**	**1.663 ± 0.476**	**12.214**	**5.276 (2.076–13.410)**	**< 0.001**
Conjunctival congestion	0.759				
Nasal congestion	**0.006**	− 1.057 ± 0.523	4.083	0.347 (0.125–0.969)	0.043
Diarrhea or bellyache	**< 0.001**	0.775 ± 0.463	2.804	2.171 (0.876–5.382)	0.094
Dizziness or headache	0.765				
Nausea or vomiting	0.051				
Sore throat	**0.006**	− 0.941 ± 0.438	4.622	0.390 (0.165–0.920)	0.032
Muscle soreness	**< 0.001**	**2.652 ± 1.000**	**7.033**	**14.187 (1.998–100.730)**	**0.008**
White blood cell count	**< 0.001**	− **0.288 ± 0.065**	**19.385**	**0.750 (0.659–0.852)**	**< 0.001**
Lymphocyte count	**< 0.001**	− 0.230 ± 0.161	2.041	0.795 (0.580–1.089)	0.153
Neutrophil cell count	**< 0.001**	0.062 ± 0.051	1.481	1.064 (0.963–1.175)	0.224
C-reactive protein level	0.300				
Chest X-ray or CT	**< 0.001**	**1.839 ± 0.192**	**91.440**	**6.291 (4.315–9.171)**	**< 0.001**

*NCP* novel coronavirus pneumonia, *β* regression coefficient, *SE* standard error, *OR* odds ratio, *CT* computed tomography.

Bold texts refer to statistical significance (*P* < 0.05).

The above characteristics were utilized in the subsequent multivariate analysis, revealing that the following nine characteristics were independent risk factors for NCP: travel or residence history within 14 days in Wuhan (OR 8.440, 95% confidence interval (CI) 4.204–16.944, *P* < 0.001), contacting patients with fever or respiratory symptoms within 14 days who had a travel or residence history in Wuhan (OR 2.967, 95% CI 1.630–5.402, *P* < 0.001), contacting patients from other areas with persistent local transmission or community with definite cases (OR 4.139, 95% CI 2.334–7.342, *P* < 0.001), relationship with a cluster outbreak (OR 25.164, 95% CI 11.833–53.516, *P* < 0.001), presence of fatigue (OR 2.710, 95% CI 1.490–4.930, *P* = 0.001), dyspnea (OR 5.276, 95% CI 2.076–13.410, *P* < 0.001), muscle soreness (OR 14.187, 95% CI 1.998–100.730, *P* = 0.008), decreased WBC count (OR 0.750, 95% CI 0.659–0.852, *P* < 0.001), and imaging changes in chest X-ray or CT (OR 6.291, 95% CI 4.315–9.171, *P* < 0.001).

### Derivation of the model

In this multivariate logistic regression model, the probability of having NCP was 1/(1 + e−(− 2.043 + 2.133 (if travelling to or residing in Wuhan) + 1.088 (if contacting patients from Wuhan) + 1.421 (if contacting patients from other areas with persistent local transmission or community with definite cases) + 3.225 (if relating to a cluster outbreak) + 0.997 (if having fatigue) + 1.663 (if having dyspnea) + 2.652 (if feeling muscle soreness) − 0.288 * WBC count + 1.839 * chest imaging score)). Consequently, we utilized the exponents of this formula and established a Zhejiang rapid screening model for predicting NCP as follows:

Model score (model 1) = 2.133 (if travelling to or residing in Wuhan within 14 days) + 1.088 (if contacting patients with fever or respiratory symptoms from Wuhan within 14 days) + 1.421 (if contacting patients with fever or respiratory symptoms from other areas with persistent local transmission or community with definite cases within 14 days) + 3.225 (if relating to a cluster outbreak) + 0.997 (if having fatigue) + 1.663 (if having dyspnea) + 2.652 (if feeling muscle soreness) − 0.288 * WBC count + 1.839 * pulmonary imaging score (as introduced in the “Method” part).

**Figure 1:**
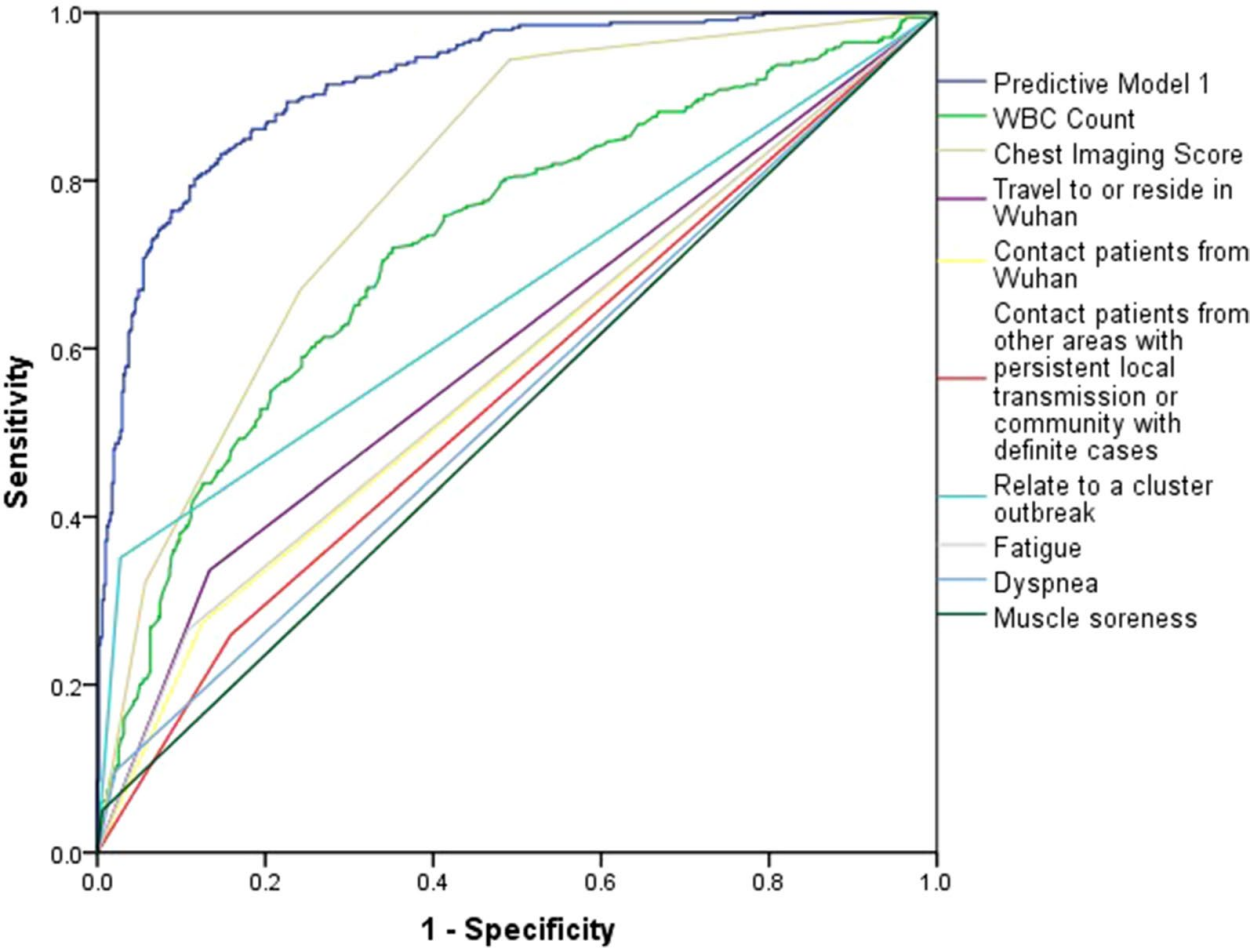
Receiver-operating characteristic (ROC) curves of the predictive model 1 and its included features for detecting novel coronavirus pneumonia. The area under the ROC curve was 0.920 (95% CI 0.902–0.938), 0.727 (95% CI 0.692–0.762), 0.795 (95% CI 0.766–0.825), with a standard error of 0.009, 0.018, and 0.015 for predictive model 1, WBC count, and chest imaging score. The optimized Youden based cutoff was 1.00, 6.20, and 0.15, respectively. The sensitivity and (1-specificity) of the binary factors were also illustrated. WBC: white blood cell.

**Figure 2:**
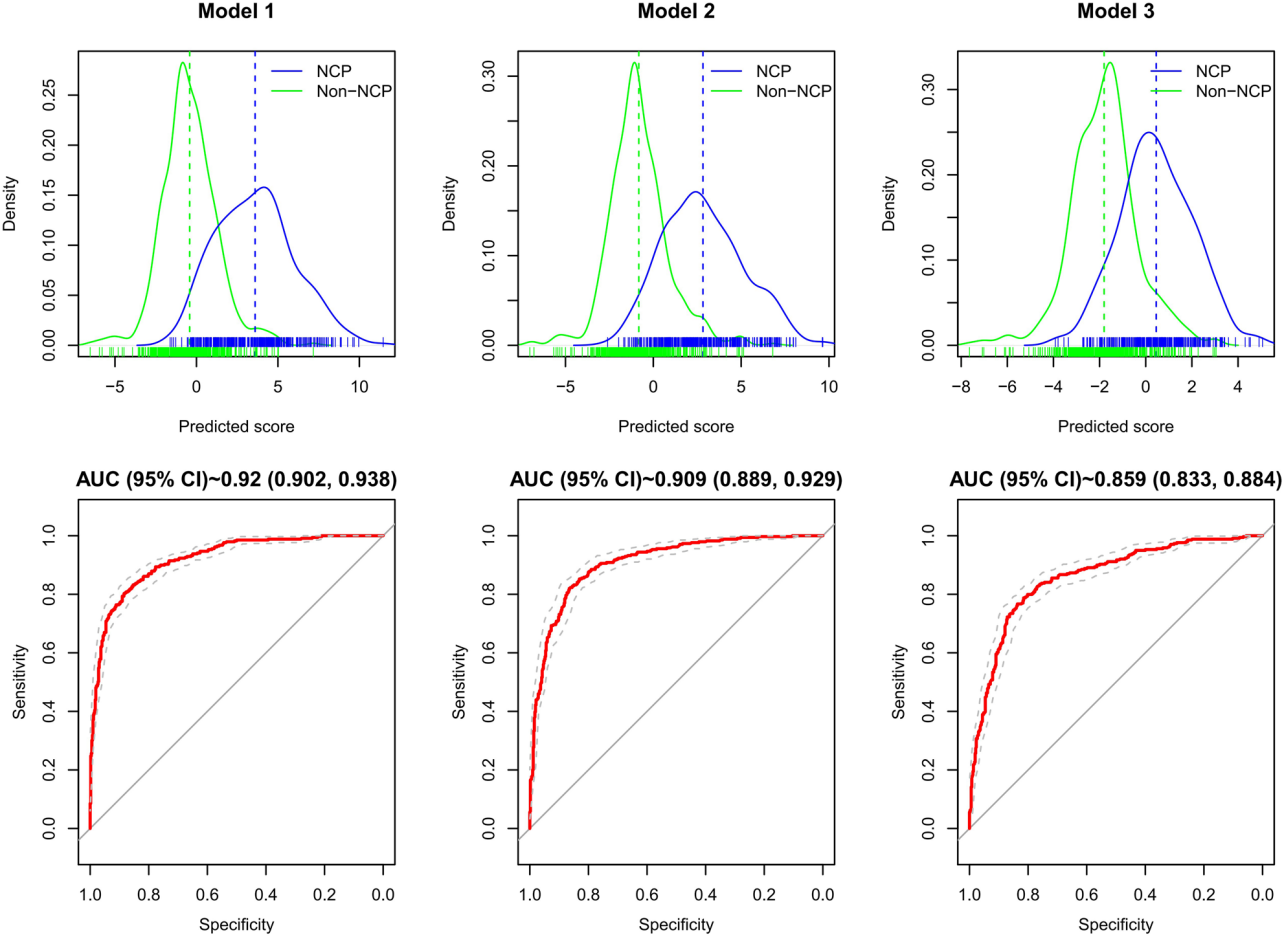
The capability of the models to discriminate novel coronavirus pneumonia. The three panels illustrate the performance of three models trained in this study. In each column the figure atop plots the fitted distribution of the predicted scores for the cases (blue) and the controls (green), respectively. The small vertical ticks underneath the distribution curve are the detailed predicted scores for individual, and the estimated mean scores of the model are presented in colored vertical lines. In the bottom plot the receiver-operating characteristic (ROC) curves (red) with the point-wise 95% confidence intervals (grey) for the corresponding prediction model. The area under the ROC curve of model 1 (the primary predictive model), model 2 (the simplified model), and model 3 (model without epidemiological history) was 0.920 (95% CI 0.902–0.938), 0.909 (95% CI 0.889–0.929), 0.859 (95% CI 0.833–0.884), with a standard error of 0.009, 0.010, and 0.013, respectively.

The AUROC of the model was 0.920 (95% CI 0.902–0.938), indicating a greater capability to discriminate NCP than WBC count (AUROC 0.727, 95% CI 0.692–0.762) or chest imaging score (AUROC 0.795, 95% CI 0.766–0.825) (Fig. 1). To internally examine whether the model was over fitted, we used fivefold Cross-Validation of the trained model, and repeated the cross-validation for 10 times. It showed that the mean of AUROC was of 0.916 with the standard deviation of 0.017. The Hosmer–Lemeshow test which measured the calibration showed a χ^2^ of 10.857 (*P* = 0.210), demonstrating that there was no significant difference from a perfect fit. The patients with NCP had a model score of 3.60 ± 2.41, higher than those without NCP (model score = −0.42 ± 1.69, *P* < 0.001) (Fig. 2). At a cut-off value of 1.0, the rapid screening model could determine NCP with a sensitivity of 85% (95% CI 81.2–88.8%), a specificity of 82.3% (95% CI 80.6–84.0%), a diagnostic accuracy of 83.2% (95% CI 80.7–85.7%), and a Youden index of 0.673.

It’s worth mentioning that at a value of whether the predicted score > 4.0, the model could detect NCP with a specificity of 98.3% (95% CI 97.7–98.9%), while the sensitivity was 42.8% (95% CI 37.6%-48.1%), and the accuracy was 76.4% (95% CI 73.5–79.2%); at a cut-off value of < −0.5, the model could rule out NCP with a sensitivity of 97.9% (95% CI 97.1–98.7%), while the specificity was 51.0% (95% CI 46.7–55.2%), and the accuracy was 69.5% (95% CI 66.4–72.6%) (Table 3). Among the 859 subjects, 154 subjects (17.9%) had a model score of > 4, and 272 subjects (31.7%) had a model score of < −0.5. Based on the cut-off values, 410 subjects (96.2% of subjects with the model score > 4 or < −0.5) were correctly classified.

**Table 3:** Diagnostic accuracy of the rapid screening model.

Cut-off value	Sensitivity (%)	Specificity (%)	LR +	LR −
− 2.0	100.0	15.2	1.18	0.00
− 1.5	99.4	22.5	1.28	0.03
− 1.0	98.8	36.2	1.55	0.03
− **0.5**	**97.9**	**51.0**	**2.00**	**0.04**
0	93.8	63.5	2.57	0.10
0.5	90.3	74.6	3.56	0.13
**1.0**	**85.0**	**82.3**	**4.80**	**0.18**
1.5	77.6	89.2	7.19	0.25
2.0	72.3	93.7	11.48	0.30
2.5	64.9	95.6	14.75	0.37
3.0	56.9	96.7	17.24	0.45
3.5	50.4	97.1	17.38	0.51
**4.0**	**42.5**	**98.3**	**25.00**	**0.58**
4.5	32.7	99.0	32.70	0.68
5.0	25.4	99.6	63.50	0.75

*LR* + positive likelihood ratio, *LR − *negative likelihood ratio.

Bold value refer to important cutoff values as addressed in the text.

### Derivation of a simplified model

We further combined all the geographical regions into one parameter as “epidemic areas with persistent local transmission or communities with definite cases”. The results of the univariate and multivariate logistic analyses were showed in Table 4. To develop a simplified model, we rounded the coefficients and elicited the model as follows.

**Table 4:** Predictors associated with NCP (combining the geographical regions).

Variables	Univariate	Multivariate			
	*P* value	β ± SE	Wald	OR (95% CI)	*P* value
Age	**< 0.001**	0.004 ± 0.007	0.355	1.004 (0.990–1.019)	0.551
Gender	0.162				
Coexisting diseases	**< 0.001**	0.495 ± 0.272	3.306	1.641 (0.962–2.798)	0.069
Travel or residence history in epidemic areas within 14 days	0.623				
Contacting patients with fever or respiratory symptoms within 14 days who had a travel or residence history in epidemic areas	**< 0.001**	**1.096 ± 0.216**	**25.703**	**2.993 (1.959–4.573)**	**< 0.001**
Relationship with a cluster outbreak	**< 0.001**	**3.018 ± 0.355**	**72.438**	**20.446 (10.205–40.967)**	**< 0.001**
Exposure to wildlife	0.092				
Contact with patients of influenza A	0.059				
Contact with patients of influenza B	0.189				
Body temperature	0.895				
Dry cough	0.052				
Sputum	**< 0.001**	0.160 ± 0.229	0.487	1.173 (0.749–1.837)	0.485
Fatigue	**< 0.001**	**0.929 ± 0.278**	**11.143**	**2.532 (1.468–4.370)**	**0.001**
Dyspnea	**< 0.001**	**1.503 ± 0.454**	**10.936**	**4.494 (1.844–10.951)**	**< 0.001**
Conjunctival congestion	0.095				
Nasal congestion	**0.006**	− **1.074 ± 0.499**	**4.627**	**0.342 (0.128–0.909)**	**0.031**
Diarrhea or bellyache	**0.001**	0.805 ± 0.428	3.533	2.237 (0.966–5.181)	0.060
Dizziness or headache	0.765				
Nausea or vomiting	**0.039**	− 0.380 ± 0.774	0.242	0.684 (0.150–3.114)	0.623
Sore throat	**0.006**	− 0.570 ± 0.416	1.882	0.566 (0.250–1.277)	0.170
Muscle soreness	**< 0.001**	**2.652 ± 0.924**	**8.242**	**14.188 (2.320–86.763)**	**0.004**
White blood cell count	**< 0.001**	− **0.293 ± 0.063**	**21.644**	**0.746 (0.660–0.844)**	**< 0.001**
Lymphocyte count	**< 0.001**	− 0.273 ± 0.171	2.549	0.761 (0.544–1.064)	0.110
Neutrophil cell count	**< 0.001**	0.057 ± 0.049	1.354	1.059 (0.961–1.167)	0.245
C-reactive protein level	0.300				
Chest X-ray or CT	**< 0.001**	**1.661 ± 0.177**	**88.133**	**5.263 (3.721–7.444)**	**< 0.001**

*NCP* novel coronavirus pneumonia, *β* regression coefficient, *SE* standard error, *OR* odds ratio, *CT* computed tomography.

Bold texts refer to statistical significance (*P* < 0.05).

Simplified model score (model 2) = 1 (if contacting patients with fever or respiratory symptoms from areas with persistent local transmission or community with definite cases within 14 days) + 3 (if relating to a cluster outbreak) + 1 (if having fatigue) + 2 (if having dyspnea) – 1 (if having nasal congestion) + 3 (if feeling muscle soreness) − 0.3 * WBC count + 2 * pulmonary imaging score. The AUROC was 0.909 (95% CI 0.889 – 0.929) (Fig. 2). In fivefold Cross-Validation, the average AUROC was 0.862, with the standard deviation of 0.028. The Hosmer–Lemeshow χ^2^ was 11.962 (*P* = 0.153). The optimal cutoff value was 0.7, with a sensitivity of 82.3% (95% CI 76.3–87.0%), a specificity of 86.2% (95% CI 82.9–88.9%), a diagnostic accuracy of 84.6% (95% CI 82.2–87.0%), and a Youden index of 0.685.

Given the evolution of the pandemic, we dropped the epidemiological history that are likely to become outdated and repeated the analysis. The risk factors and their ORs were exhibited in Table 5. Consequently, a predictive model without epidemiological history was established.

**Table 5:** Predictors associated with NCP (dropping the epidemiological history).

Variables	β ± SE	Wald	OR (95% CI)	*P* value
Age	0.001 ± 0.006	0.047	1.001 (0.989–1.014)	0.828
Coexisting diseases	**0.556 ± 0.242**	**5.297**	**1.744 (1.086–2.802)**	**0.021**
Sputum	0.047 ± 0.203	0.054	1.048 (0.704–1.561)	0.816
Fatigue	**0.813 ± 0.247**	**10.826**	**2.255 (1.389–3.660)**	**0.001**
Dyspnea	**1.238 ± 0.426**	**8.436**	**3.448 (1.496–7.948)**	**0.004**
Nasal congestion	− 0.751 ± 0.420	3.187	0.472 (0.207–1.076)	0.074
Diarrhea or bellyache	0.763 ± 0.393	3.771	2.144 (0.993–4.629)	0.052
Nausea or vomiting	− 0.058 ± 0.660	0.008	0.944 (0.259–3.445)	0.931
Sore throat	− 0.404 ± 0.345	1.374	0.667 (0.339–1.312)	0.241
Muscle soreness	**2.355 ± 0.883**	**7.119**	**10.538 (1.868–59.434)**	**0.008**
White blood cell count	− **0.315 ± 0.059**	**27.946**	**0.730 (0.650–0.820)**	**< 0.001**
Lymphocyte count	− **0.303 ± 0.152**	**3.993**	**0.738 (0.548–0.994)**	**0.046**
Neutrophil cell count	0.042 ± 0.047	0.814	1.043 (0.952–1.143)	0.367
Chest X-ray or CT	**1.582 ± 0.155**	**104.042**	**4.863 (3.589–6.590)**	**< 0.001**

*NCP* novel coronavirus pneumonia, *β* regression coefficient, *SE* standard error, *OR* odds ratio, *CT* computed tomography.

Bold texts refer to statistical significance (*P* < 0.05).

Model score without epidemiological history (model 3) = 0.6 (if having coexisting diseases) + 0.8 (if having fatigue) + 1.2 (if having dyspnea) + 2.4 (if feeling muscle soreness) − 0.3 * WBC count − 0.3 * Lymphocyte count + 1.6 * pulmonary imaging score. The AUROC was 0.859 (95% CI 0.833–0.884) (Fig. 2), with the optimal cutoff value of − 1, a sensitivity of 83.5% (95% CI 79.1–87.1%), a specificity of 76.0% (95% CI 72.1–79.4%), a diagnostic accuracy of 78.9% (95% CI 76.2–81.7%), and a Youden index of 0.595. Repeated fivefold Cross-Validation showed the average AUROC was 0.854, with the standard deviation of 0.027. The Hosmer–Lemeshow χ^2^ was 12.218 (*P* = 0.142), indicating no statistical difference from a perfect fit.

## Discussion

In this study, we compared the characteristics between the NCP patients and the suspected individuals who were finally ruled out of NCP. Having analyzed the clinical and epidemiological features, we developed a rapid screening model for predicting NCP in a Zhejiang population. The model included four epidemiological features: travel or residence history within 14 days in Wuhan, contacting patients with fever or respiratory symptoms within 14 days from Wuhan, contacting patients from other areas with persistent local transmission or community with definite cases, relationship with a cluster outbreak; and five clinical manifestations: fatigue, dyspnea, muscle soreness, decreased WBC count, and imaging changes in chest X-ray or CT. The diagnostic performance of the established scale was excellent with an AUROC of 0.920.

At a cut-off value of > 1.0, the model could detect NCP with a sensitivity of 85% and a specificity of 82.3%. Due to the nature of a communicable disease, the associated costs of a false negative are huge, therefore it is essential to avoid missed diagnoses, in particular given its surging outbreak. When the score was higher than 4.0, subjects were more likely to suffer from NCP (with a specificity of 98.3%) and they should be immediately isolated and further tests are highly recommended. In contrast, during the outbreak, a great quantity of patients with flu-like symptoms were scared and crowded into hospitals, giving clinicians great pressure. A model score of < −0.5 demonstrated a very small probability to be infected by 2019-nCoV (with a sensitivity of 97.9%). Clinicians can set the best cut-off value based on actual demands.

Under the circumstance of continuing spread of 2019-nCoV, Zhejiang model established in this study, as the first rapid screening diagnostic model for NCP, is of great significance in this battle. Unlike virus isolation or RT-PCR testing, the screening scale is economical, uncomplicated and fast, which can be used to select potential patients for further RT-PCR examinations.

Nevertheless, there were several limitations based on the model. Firstly, the enrolled participants were limited to Zhejiang Province, leading to certain regional limitations in the application of the screening model, in particular the epidemiological characteristics of possible compromise in another area. Secondly, our research was confined to early and rapid screening, without adequate information on disease progression and prognosis. Last but not least, with the development of epidemic situation, weight of certain characteristics, especially epidemiological characteristics, should be modified to increase the scope and accuracy of the diagnostic model.

In the purpose of eliminating the effects of location-specific factors like Wuhan-related criteria, which might be no longer applicable with the evolution of the pandemic, a simplified model was developed by combining all the epidemic regions. Furthermore, we repeated the analysis by dropping the epidemiological data. Both of the subsequent models were proved to be effective. In addition, fivefold Cross-Validations were repeated in each model during internal validation to quantify any optimism in the predictive performance, and Hosmer–Lemeshow χ^2^ test was utilized to measure calibration. Further nationwide even worldwide studies are needed to access the utility of this model, and subject to further adjustment and calibration if necessary.

According to the recent literatures, most patients with NCP are characterized by fever, cough, fatigue, and myalgia in the initial stage^11^. Atypical symptoms include diarrhea, nausea, headache, sore throat and so on. As the illness progressed, a proportion of patients gradually presented with dyspnea, especially in the populations with low immune functions^12^. Complications like acute respiratory distress syndrome (ARDS), arrhythmia and shock, is probably associated with a poor prognosis^7^.

The most common laboratory abnormalities observed are leukopenia and lymphocytopenia. Moreover, it is reported that hypoalbuminemia, elevated CRP and lactate dehydrogenase (LDH), and decreased CD8 count can be seen in part of cases^6,13^. The most frequent imaging manifestation is patchy/punctate ground glass opacities involved in single or multiple pulmonary lobes^14^. Alterations on chest CT can reflect the severity and progress of NCP^15^. However, 2019-nCoV infection can also present with normal pulmonary imaging, particularly in early stage, suggesting the necessity to combine epidemiological information, clinical manifestations and imaging in the screening and diagnosis^16^.

At present, RT-PCR remains the confirmation criteria for the diagnosis of 2019-nCoV infection. RT-PCR is a technology combining RNA reverse transcription (RT) with polymerase chain amplification (PCR) of cDNA. It has been widely used in detecting different coronavirus (such as SARS-CoV and MERS-CoV) in laboratory, because of its high specificity and sensitivity. Besides that the RT-PCR test can be time-consuming, a shortage of test kits supply may not meet the needs of a growing infected population. Furthermore, RT-PCR of 2019-nCoV may be false negative due to unstable kits or unstandardized sampling^17^. Xiao et al. reported that some patients who met the diagnosis of NCP based on clinical and imaging findings, had negative results for viral RNA^18^. In another study, initial negative RT-PCR results turned positive in repeated testing in a number of patients^10^. With the purpose of timely isolation and early treatment, it is necessary to establish a rapid screening diagnostic model for distinguishing highly suspicious patients with NCP. Actually, the authors are trying to develop a procedure for fast scoring in clinical application based on this model.

In conclusion, the study established a rapid screening model for predicting NCP in a Zhejiang population. What’s more, we developed a simplified model by combining the epidemic regions and rounding the coefficients, as well as a model without any epidemiological factor. The models can be used as a simple, fast, and cost-effective tool for screening NCP with significant clinical value.

## Methods

### Patients

From January 17 to February 19, 2020, a total of 880 patients who were suspected of 2019-nCoV infection were recruited from hospitals in Hangzhou, Wenzhou, Shaoxing, Taizhou, Ningbo and Jiaxing in Zhejiang Province. The study was approved by the Ethics Committee of Zhejiang Provincial People’s Hospital (2020KY006); in addition, all research was performed in accordance with relevant guidelines^19^. Exempt informed consent was approved by the Ethics Committee of Zhejiang Provincial People’s Hospital because the subjects would not be exposed to any risk in this observational study, and the information of subjects was anonymized at collection and prior to analysis.

Epidemiological history and clinical manifestations were collected in each individual. Age, gender, region, coexisting diseases, body temperature, results of blood routine test and chest X-ray or CT were recorded for all participants. Throat swab, sputum, blood, or stool samples were collected to examine the 2019-nCoV nucleic acid using real-time RT-PCR. If the first-time RT-PCR test revealed negative, samples should be collected after 24 h for a repeated test.

### Eligibility

Patients admitted to the fever clinics who were initially suspected of NCP were included in the study. Suspected or confirmed cases were diagnosed according to the 5th edition of the Chinese recommendations for diagnosis and treatment of pneumonia caused by 2019-nCoV^19^.

#### Suspected cases

NCP should be suspected if subjects conform to any one of the criteria in the epidemiological history and any two of the standards in clinical presentations. If there is no epidemiological history, suspected cases should meet three of the criteria in clinical presentations.

Epidemiological history: (1) Subjects with a travel or residence history in Wuhan or its neighboring areas, or other areas with persistent local transmission, or communities with definite cases within 14 days; (2) Subjects with a history of contacting confirmed cases with 2019-nCoV infections (positive nucleic acid detection) within 14 days; (3) Subjects with a history of contacting patients with fever or respiratory symptoms who have a travel or residence history in Wuhan or its neighboring areas, or in other areas with persistent local transmission, or communities with definite cases within 14 days; (4) Subjects who are associated with a cluster outbreak, which is defined as one definite case with NCP in family or place of work within 14 days, along with other patients with fever or respiratory symptoms.

Clinical presentations: (1) Fever and/or respiratory symptoms; (2) Typical chest imaging features of NCP, such as ground-glass opacity, infiltrating shadows, and pulmonary consolidation. (3) Normal or decreased white blood cell (WBC) count, or decreased lymphocyte count in the early stage of the disease.

#### Confirmed cases

Suspected cases who accord with any one of the following criteria: (1) Positive 2019-nCoV nucleic acid in throat swab, sputum, blood samples, or stool by using real-time RT-PCR; (2) Genetic sequencing of samples being highly homologous with the known 2019-nCoV.

### Establishment of the rapid screening scale

Based on the epidemic situation in Zhejiang Province, we included age, gender, co-existing diseases, the epidemiological parameters, clinical symptoms, body temperature, WBC count, lymphocyte count, neutrophil count, and chest imaging to establish a novel diagnostic model of NCP. The epidemiological features and symptoms were considered as binary variables, and were scored as “1” if “yes”, and “0” if “no”. As to chest radiologic changes, they were simply classified as “normal”, “unilateral local patchy shadowing”, “bilateral multiple ground glass opacity”, “bilateral diffuse ground glass shadowing with pulmonary consolidation”, and “Other imaging alterations such as pulmonary nodule or pleural effusion”, and were scored as “0”, “0.5”, “1”, “2” and “0.3”, respectively.

The samples were classified to NCP, 339 individuals, and non-NCP, 520 individuals, according to their real-time PT-PCR outcomes since the detection of the 2019-nCoV nucleic acid using real-time RT-PCR was considered the golden standard. Consequently, after the derivation of the screening model, the diagnostic performance of the established scale was also verified. We further combined all the geographical regions into one parameter, that is “epidemic areas with persistent local transmission or communities with definite cases”, and developed a simplified model by rounding the coefficients. Moreover, we dropped the epidemiological parameters that are likely to become outdated given the evolution of the pandemic, and repeated the analysis.

### Statistical analysis

Statistical analyses were conducted using SPSS software (version 22.0) for Windows (SPSS, Chicago, IL). Continuous variables were presented as mean ± standard deviation. Continuous variables were compared using the Student’s t-test, and categorical variables were compared using the chi-squared test. For multiple comparisons, the one-way analysis of variance (ANOVA) was performed. Univariate logistic regression analyses were conducted to assess the factors associated with NCP. The parameters with statistical significance were loaded to a multivariate logistic regression model to further identify independent predictors for NCP.

To identify candidate predictors, we performed a stepwise logistic regression analysis (*P* value to enter = 0.05 and *P* value to remove = 0.10). A model based on the results of multiple logistic regression analysis was established to screen NCP; furthermore, fivefold cross-validation was employed repeatedly for 10 times to evaluate the performance of the model and examine whether the model was over fitted. Model calibration was evaluated using the Hosmer–Lemeshow χ^2^ test. Area under receiver operating characteristic curve (AUROC) with 95% CI was used to assess the predictive accuracy of the screening model for determining NCP^20^. Bootstraps with 500 resample were applied to overplot the point-wise 95% CIs of the ROC curves by the R software (version 4.0.3) (not to re-estimate the regression coefficients). Optimal cut-off values were set, and the corresponding sensitivities, specificities, diagnostic accuracies, positive likelihood ratios, and negative likelihood ratios of the model were calculated. A two-sided *P* value cutoff < 0.05 was considered to be statistically significant.

## References

[1] Lu R (2020). Genomic characterisation and epidemiology of 2019 novel coronavirus: implications for virus origins and receptor binding. Lancet.

[2] Chan JF (2020). A familial cluster of pneumonia associated with the 2019 novel coronavirus indicating person-to-person transmission: a study of a family cluster. Lancet.

[3] Li Q (2020). Early transmission dynamics in Wuhan, China, of novel coronavirus-infected pneumonia. N. Engl. J. Med..

[4] Tuite AR, Fisman DN (2020). Reporting, epidemic growth, and reproduction numbers for the 2019 novel coronavirus (2019-nCoV) epidemic. Ann. Intern. Med..

[5] Update on the outbreak of new coronavirus pneumonia as at 24 February 27. http://www.nhc.gov.cn/xcs/yqtb/202002/d5e15557ee534fcbb5aaa9301ea5235f.shtml. (assessed on February 27th, 2020).

[6] Huang C (2020). Clinical features of patients infected with 2019 novel coronavirus in Wuhan, China. Lancet.

[7] Wang D (2020). Clinical characteristics of 138 hospitalized patients with 2019 novel coronavirus-infected pneumonia in Wuhan, China. JAMA.

[8] Chen Z (2020). Diagnosis and treatment recommendations for pediatric respiratory infection caused by the 2019 novel coronavirus. World J. Pediatr..

[9] Corman V (2020). Detection of 2019 novel coronavirus (2019-nCoV) by real-time RT-PCR. Euro. Surveill..

[10] Xie X (2020). Chest CT for typical 2019-nCoV pneumonia: relationship to negative RT-PCR testing. Radiology.

[11] Xu XW (2020). Clinical findings in a group of patients infected with the 2019 novel coronavirus (SARS-Cov-2) outside of Wuhan, China: retrospective case series. BMJ.

[12] Chen N (2020). Epidemiological and clinical characteristics of 99 cases of 2019 novel coronavirus pneumonia in Wuhan, China: a descriptive study. Lancet.

[13] Liu Y (2020). Clinical and biochemical indexes from 2019-nCoV infected patients linked to viral loads and lung injury. Sci. China Life Sci..

[14] Pan Y (2020). Initial CT findings and temporal changes in patients with the novel coronavirus pneumonia (2019-nCoV): a study of 63 patients in Wuhan, China. Eur. Radiol..

[15] Pan F (2020). Time course of lung changes on chest CT during recovery from 2019 Novel Coronavirus (COVID-19) Pneumonia. Radiology.

[16] Chung M (2020). CT imaging features of 2019 novel coronavirus (2019-nCoV). Radiology.

[17] Hao W, Li M (2020). Clinical features of atypical 2019 novel coronavirus pneumonia with an initially negative RT-PCR assay. J. Infect..

[18] Xiao SY, Wu Y, Liu H (2020). Evolving status of the 2019 novel coronavirus infection: proposal of conventional serologic assays for disease diagnosis and infection monitoring. J. Med. Virol..

[19] China National Health Commission. Diagnosis and treatment of pneumonitis caused by new coronavirus (trial version 5). Beijing: China National Health Commission, 2020. http://www.nhc.gov.cn/yzygj/s7653p/202002/d4b895337e19445f8d728fcaf1e3e13a.shtml (accessed February 8, 2020).

[20] Swets JA (1988). Measuring the accuracy of diagnostic systems. Science.

